# Transvaginal natural orifice transluminal endoscopic surgery (vNOTES) as treatment for upper vaginal leiomyoma: A case report

**DOI:** 10.1097/MD.0000000000025969

**Published:** 2021-05-21

**Authors:** Jian-Hong Liu, Ying Zheng, Ya-Wen Wang

**Affiliations:** a Department of Obstetrics and Gynecology, West China Second Hospital, Sichuan University; b Key Laboratory of Birth Defects and Related Disease of Women and Children, Sichuan University, Ministry of Education, People's Republic of China.

**Keywords:** leiomyoma, minimally invasive surgery, myomectomy, vagina, vNOTES

## Abstract

**Introduction:**

Transvaginal natural orifice transluminal endoscopic surgery (vNOTES) is an emerging technique in the area of minimally invasive surgery. Vaginal leiomyoma is a rare benign tumor, with only a few cases being reported in the literature. we demonstrate a novel approach for excision of a vaginal leiomyoma via vNOTES. To ensure reproducibility and replicability akin to a standardized procedure, we have provided a step-by-step video description of the use of vNOTES for upper anterior vaginal myomectomy.

**Patient concerns:**

A 35-year-old female (G2P0A2) presented with a tumor in the upper anterior vaginal wall, which gradually increased in size.

**Diagnosis:**

A vaginal examination revealed a swollen area approximately 3-cm in diameter on the upper anterior vaginal wall. The swelling was mobile and solid. All other vitals were normal. Transvaginal ultrasound detected a 3.0 × 3.4 cm hypoechogenic mass on the superior vaginal wall, and a preoperative diagnosis of the vaginal tumor was confirmed.

**Interventions:**

The upper vaginal leiomyoma treated using transvaginal natural orifice transluminal endoscopic surgery.

**Outcomes:**

The procedure lasted for 20 min, and the postoperative course was uneventful.

**Conclusions:**

vNOTES can be a promising alternative to traditional vaginal surgery for upper vaginal disease due to advantages such as excellent exposure, easy access and precise suturing. However, more studies are needed to assess its long-term efficacy.

## 1 Introduction

Vaginal leiomyoma is a rare benign tumor, with only a few cases described in the literature.^[1–3]^ Depending on the size and site, most patients with vaginal leiomyoma have no clinical symptoms. However, patients with large leiomyoma may have varied clinical symptoms such as pain, dyspareunia, or labor dystocia. The definitive treatment for large leiomyoma is surgical removal and subsequent histological evaluation to confirm the diagnosis to rule out malignancy. Surgery for removal of vaginal leiomyoma can either be performed via the abdominal or vaginal route depending on the tumor's anatomic location. A transvaginal approach is the most favored approach and is used in 90%. In comparison, only 10% of vaginal leiomyomas are resected via transabdominal approach, with or without concomitant hysterectomy, especially if the tumor is large and located in the upper part of the vagina.^[4]^

Transvaginal natural orifice transluminal endoscopic surgery (vNOTES) has emerged as a minimally invasive technique in gynecological surgery in the recent years. It has been used successfully in gynecologic surgeries such as salpingectomy, myomectomy, ovarian cystectomy, hysterectomy, tubal reversal, sacrocolpopexy and lymphadenectomy, its clinical indications are currently being explored.^[5–7]^

Here, we report a case of leiomyoma that occurred in the upper anterior vaginal wall, resected successfully using vNOTES, which may serve as an alternative to traditional surgery for upper vaginal disease. We examine our case against 9 previously reported cases that used a similar approach to resolve various vaginal conditions to understand the benefits and stability of this technique.

## 2 Case report

A 35-year-old female (G2P0A2) presented with a tumor in the upper anterior vaginal wall. The mass was detected two years ago and had been growing larger gradually. The patient did not experience any symptoms, including dysuria or dyspareunia with no associated pain or tenderness. The patient had regular menstrual periods and had no relevant family history. Vaginal examination revealed a swollen area approximately 3-cm in diameter on the upper anterior vaginal wall. The swelling was mobile and solid. Mucosa over the swelling appeared to be healthy. Routine laboratory tests, including routine blood test, coagulation test, biochemistry profile, and tumor marker levels were within the normal reference ranges. Transvaginal ultrasound detected a 3.0 × 3.4 cm hypoechogenic mass on the superior vaginal wall, and a preoperative diagnosis of vaginal tumor was confirmed. Following a full preoperative evaluation, vaginal mass resection via vNOTES was decided. Written informed consent was obtained from the patient for publication of this article and the accompanying video.

**Figure 1:**
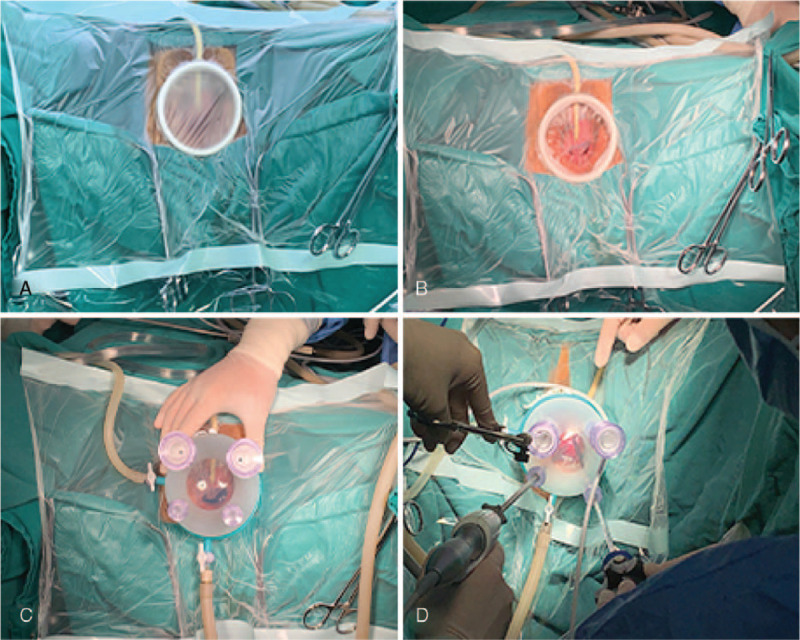
Setting of the transvaginal multichannel single-port for good sealing. The patient was shifted to a lithotomy position after general anesthesia. After disinfection, towels and wound retractor were placed, and a neurosurgical operative membrane was spread (A). The inner part of the neurosurgical membrane was removed along the edge of the wound retractor (B). using the neurosurgical membrane to achieve a good trans-vaginal insufflation (C–D).

**Figure 2:**
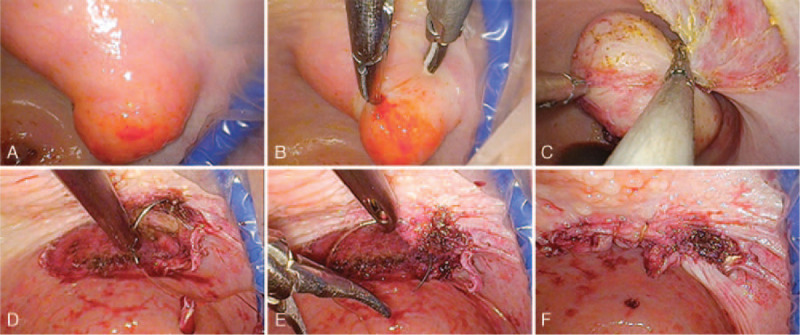
vNOTES myomectomy procedure. The location of the cervix and vaginal tumor via vNOTES (A). We used traditional laparoscopic instruments to resect the vaginal tumor (B-C). The vaginal wound was closed using continuous sutures (D–F).

The video clearly shows the setting of the transvaginal multichannel single-port for good sealing and the detailed procedure of vaginal myomectomy via vNOTES (see Video, Supplemental Video (Video that demonstrates the use of vNOTES for upper anterior vaginal myomectomy, 5 min, 40 s, 371MB.), which demonstrates the use of vNOTES for upper anterior vaginal myomectomy):

1. Cystoscopy was performed, and bilateral ureteral stents were placed at the beginning of the surgery. The surgery was performed in the lithotomy position while the patient was under general anesthesia. Since the vaginal mass was closely associated with urinary system, a flexible cystoscopy was performed to check the urinary bladder and ureteral orifices. If no abnormalities were detected, bilateral ureteral stents were placed to ensure safety.
2. Setting of the transvaginal multichannel single-port for maintaining a stable trans-vaginal insufflation. The vaginal borders became smoother after CO_2_ insufflation. We used 14 mm Hg of pressure for trans-vaginal insufflation with 7 L/min flow for maintenance. The most complex part of this surgery was obtaining a good seal. Successful establishment of a stable trans-vaginal insufflation is key to the success of the surgery. In this video, we clearly show our tips and tricks (use of neurosurgical operative membrane) to establish a stable trans-vaginal insufflation (Fig. 1).
3. Vaginal mass was excised using laparoscopic resection techniques. An incision was made on the most prominent part of the swelling, and the mass was dissected away from all the surrounding tissue. As an advantage of the vNOTES procedure, the boundary between the vaginal wall and the surrounding organs could be clearly seen intraoperatively (Fig. 2). The excised mass showed a typical whorled pattern suggestive of vaginal leiomyoma and was sent for histopathology.
4. Histology showed a vaginal leiomyoma. The resected mass was subsequently identified as vaginal leiomyoma.
5. Closure of the vaginal wound was performed with continuous sutures after checking for hemostasis (Fig. 2). To facilitate suturing, we used a 3–0 absorbable barbed suture.
6. Cystoscopy was performed again to ensure there was no injury, and bilateral ureteral stents were pulled out.

The upper anterior vaginal myomectomy via vNOTES was successful with a relatively easy gesture; no intraoperative or postoperative complications occurred. The surgery lasted 20 min, with an estimated blood loss of 5 mL. The patient was discharged 5 h after surgery; her postoperative course was painless. At follow-up 3 months later, she was asymptomatic, and bimanual examination revealed a soft anterior vaginal wall.

## 3 Discussion

We described a case of upper vaginal leiomyoma treated using transvaginal natural orifice transluminal endoscopic surgery. Our findings reveal that resection of the upper vaginal leiomyoma via vNOTES is feasible and effective for high-skilled surgeons with experience in performing laparoendoscopic single-site surgery. We believe that for a tumor in the upper anterior wall of the vagina, as well as in obese patients with a deep vagina, exposure to the surgical field is challenging. Vaginal myomectomy via vNOTES as against traditional vaginal surgery can offer a clearer approach and facilitate a better outcome.

vNOTES is an emerging and promising minimally invasive approach for gynecological diseases. We reviewed the literature on vNOTES for various vaginal conditions (Table 1).^[8–11]^ The use of a similar technique has been described for treating eroded and/or infected sacrocolpopexy mesh and incomplete longitudinal vaginal septum in the published literature. There were 9 published cases, including 7 cases of vaginal mesh infection and erosion. Notably, all cases were successfully managed without any complications suggesting that vNOTES may be a viable option for the treatment of various vaginal complications, such as vaginal mesh erosion, vaginal septum, and upper vaginal leiomyoma.

**Table 1.** Summary of reports on vaginal conditions treated with transvaginal natural orifice transluminal endoscopic surgery.

First author, year	Study size	Patient age (year)	Disease	Symptom	Interventions	Operation time	Complications	Discharge time
Fernando Heredia et al [2019]^[9]^	2	35	incomplete longitudinal vaginal septum	Dyspareunia	complete resection of the septum	5 min	no	4 h
		36	a 3-cm leiomyoma in the proximal vaginal third	Dyspareunia	vaginal myomectomy	35 min	no	12 h
Valentina Billone et al [2015]^[8]^	5	Unknown	vaginal mesh erosion	mesh erosion	mesh excision	unknown	no	the same day
Stefan Mohr et al [2017]^[10]^	1	68	vaginal mesh infection and erosion	foul-smelling vaginal discharge	abscess irrigation and mesh excision	unknown	no	unknown
Marie Schaub et al [2017]^[11]^	1	59	recurrent mesh infection and erosion	foul-smelling vaginal discharge	abscess irrigation and mesh excision	60 min	no	unknown

Transvaginal single-port laparoscopic instruments for vaginal surgery showed two main advantages:

1. excellent exposure of the affected area and safe operation, especially for tumors located on the upper anterior vaginal wall, and
2. easy access and precise suturing.

We described a new application of vNOTES, with a novel way to resolve upper vaginal complications. For surgeons trained in vNOTES, this technique may offer a more ergonomic approach than the traditional vaginal route and offer improved visualization of the target area.

As a negative aspect, this technique has a steep learning curve. The surgeons, in our surgical team, were highly experienced and have performed 5000+ single-port surgeries. Our team believes that doctors can safely and successfully perform vNOTES if they explore single-port technology in-depth and master the basic surgical skills related to single-port surgery. We believe that more case reports and studies are needed to assess the long-term outcomes of our treatment approach.

## Acknowledgments

The authors would like to thank the patient for agreeing to reveal the case details for publication.

## Author contributions

**Conceptualization:** Jian-Hong Liu, Ya-Wen Wang.

**Investigation:** Jian-Hong Liu.

**Methodology:** Jian-Hong Liu.

**Resources:** Ying Zheng.

**Supervision:** Ying Zheng, Ya-Wen Wang.

**Validation:** Jian-Hong Liu.

**Visualization:** Jian-Hong Liu.

**Writing – original draft:** Jian-Hong Liu.

**Writing – review & editing:** Ying Zheng.

## Supplementary Material

Supplemental Digital Content
